# Adaptation and validation of the Longer-term Unmet Needs after Stroke (LUNS) monitoring tool in Sri Lanka

**DOI:** 10.1186/s12889-023-16636-1

**Published:** 2023-09-04

**Authors:** Nalinda Tharanga Wellappuli, Hettiarachchige Subashini Rasanja Perera, Gunendrika Kasthuriratne, Thashi Chang, Nalika Sepali Gunawardena

**Affiliations:** 1 Centre for Health Economics and Policy Innovation (CHEPI), Imperial College London, London, England; 2 Ministry of Health, ‘Suwasiripaya’, No-385, Rev. Baddegama Wimalawansa Thero Mawatha, Colombo-10, Sri Lanka; 3 Department of Rheumatology and Rehabilitation, National Hospital, E.W. Perera Road, Colombo-10, Sri Lanka; 4 Department of Clinical Medicine, Faculty of Medicine, University of Colombo, No- 25, Kynsey Road, Colombo 08, Sri Lanka; 5 World Health Organization-Southeast Asia Regional Office, New Delhi, India

**Keywords:** Stroke, Sri Lanka, Long-term care, Needs, Health services, Factor analysis, Delphi technique

## Abstract

**Background:**

Globally, stroke is a leading cause of mortality and morbidity. Unmet needs are defined as expressed needs that are not fulfilled by services provided and are considered an important indicator of the adequacy and quality of stroke follow-up care. This study aimed to culturally adapt, modify, translate and validate, the Longer-term Unmet Needs after Stroke (LUNS) monitoring tool, to Sri Lanka. Currently, there is no validated tool in Sri Lanka to assess unmet needs among stroke survivors and unmet needs are not systematically assessed.

**Methods:**

A phased approach followed to culturally adapt, translate, establish its factorial validity and evaluate the convergent and divergent validity, reliability, and overall acceptability. The process of culturally adapting the tool was carried out using two rounds of the modified Delphi technique. The modified tool was translated to Sinhala and pretested among 10 stroke survivors. A descriptive cross-sectional study was conducted among 119 stroke survivors to establish the factorial validity and convergent and discriminant validity using the GHQ-12 and Barthel Index. The Socio-demographic characteristics of the study participants are presented. Communalities were assessed for 21 items and 2 items were dropped. Factor structure was confirmed with varimax and oblique rotations. The correlation coefficient was calculated to assess convergent and divergent validity. Cronbach’s alpha value was calculated to assess internal reliability.

**Results:**

Following the modified Delphi technique, 5 items of LUNS tool were removed, and 5 items were modified. Three new items were added based on expert recommendation. One item related to driving also removed as it does not fit with the factor structure emerged. In establishing factorial validity 5 factors emerged from the exploratory factor analysis. In assessing the convergent and discriminant validity, test results revealed that both General Health Questionnaire-12 (GHQ-12) and Barthel Index significantly correlated as expected with unmet needs. The results of Cronbach’s alpha showed that all the factors were moderately high confirming the reliability of the tool.

**Conclusions:**

The Sinhala version of the LUNS monitoring tool is a valid and reliable instrument to assess the unmet needs of stroke survivors. Assessment of unmet needs will add new insight into evaluation of the quantity, quality, and effectiveness of healthcare interventions received by stroke survivors in Sri Lanka.

## Background

Stroke is identified as the second highest ranked disease to cause disability adjusted life years [1] and also ranked the second highest cause of death globally in the year 2019, [2] affecting both developed countries as well as developing countries. In Sri Lanka, the cerebrovascular disease was the seventh leading cause of hospital mortality with 2396 deaths reported within state healthcare institutions totalling 7.6% of proportionate mortality in the year 2019 [3]. Estimates of incidence or prevalence of stroke in Sri Lanka are scarce with the latest estimate of prevalence being 1% by a community survey conducted in the Colombo district in the year 2013 among 2313 adults [4]. Additionally, the National Survey on Self-reported Health among 25,000 households in Sri Lanka in 2014 revealed that the prevalence of stroke/paralysis among the population aged 15 and above was 0.5% [5].

Stroke affects bodily functions limiting the performance of activities and participation. The main disabilities are related to walking, speaking, continence, cognition, swallowing, vision, and social participation [6]. Following the acute stage of stroke, the survivors need to be followed up to manage physical and mental health needs [7, 8] and healthcare professionals use an array of clinical tools in assessing the improvement of the health status.

One other important aspect of follow-up care is assessing and attending to the unmet needs of stroke survivors. Unmet needs are defined as ‘expressed needs that are not fulfilled by services provided’ [9]. Unmet needs among stroke patients may be a complex phenomenon resulting from unsatisfactory care received related to the physical, mental and social needs of stroke survivors. Globally, there are only a few tools that have been developed with statistical validation to assess the unmet needs among stroke survivors [10]. The Southampton needs assessment questionnaire (SNAQ) with 77 questions was validated using content validity in the first step [11]. They reported a predictive validity as expected for the study population, but they calculated Chi Squares in assessing discriminant validity and reported as the interpretations were not straightforward. They also confirmed convergent validity as the number of unmet needs was neither related to their level of disability as scored by the OPCS scale (Spearmen *r*= -0.03), nor their FIM scores (*r* = 0.11). They also measured internal reliability using nonparametric tests but did not report any other values.

The Self-Reported Long-Term Needs After Stroke study developed an unmet needs assessment tool by using the Medical Research Council general practice stroke register based on the previous questionnaires [12]. This tool consisted of 44 closed questions related to information about stroke, health after stroke, everyday living, work and leisure, friends, family and use of support groups, finances and demographic information. Even though the preliminary version of the tool was tested and reviewed by a patient and family group none of the psychometric properties or any other tool validation related information was reported. They have estimated and presented unmet needs in the form of frequencies and proportions. This tool also adapted and used in Australia with version of questions 30 and 58 and in Ireland 49 questions but none reported any psychometric properties in adaptation [13].

The LUNS tool is a 22 item- validated tool developed in the United Kingdom among 770 stroke survivors living at home for 3 to 6 months following stroke [14]. This study conducted two phases with questionnaires posted to the selected participants for the study. The content of the tool is in areas of information needs, service needs, emotional and social consequences and health problems and related issues. The study team used GHQ-12 to measure concurrent validity and reported significant difference in health status between those who did and did not identify unmet needs (*p* < 0.05). They also reported, significantly poorer health status among the groups with unmet needs compared to the no unmet need group which was assessed using SF-12 questionnaire, Frenchy Activities index (*P* < 0.05). The test and retest reliability also reported across 22 items with Kappa statistics ranging from (0.673 to 0.445) and percentage agreement ranging from 95.8% to 85.7%. The Cronbach’s efficient alpha was assessed to measure the internal consistency and it was 0.815. They reported LUNS tool may consist of possible 4 dimensions but did not report dimensions [15]. In year 2017 this tool translated and culturally adapted and validated to use in Netherlands [16]. They validated tool among hospital base stroke survivors of 5–8-years duration and 78 survivors responded to the questionnaire. The median number of unmet needs was 3.5 among the study population and 15 out of 22 items had a significant association with FAI or SF-12 mental or physical component.

In Sri Lanka, the assessment of unmet needs among stroke survivors has not been systematically studied. Assessment of the unmet needs of stroke survivors will add new insight into the evaluation of the quantity, quality and effectiveness of healthcare interventions received by stroke survivors in Sri Lanka as it incorporates patients’ perspective on the impact of medical and care decisions on their physical, mental and social needs.

We conducted this study to culturally adapt, modify, and translate into the Sinhala language and validate the Longer-term Unmet Needs after Stroke (LUNS) monitoring tool. We selected this tool based on the factors such as the process of statistical validation of development of the tool, the relatively easy response with Yes/No answers, and the quick time in completion of the tool compared to other tools as well. Clinically defined outcomes may be limited in use in assessing long-term needs after stroke. We believe the inclusion of multiple types of needs within the tool also help the multidisciplinary team in adjusting the follow up care for individual stroke survivors. We hypothesized this instrument is a valid and reliable tool to assess the unmet needs of stroke survivors and we conducted this study to assess it.

## Methods

### The LUNS tool

The 22 item LUNS version 1-2008 is a tool that assesses the unmet needs of stroke victims comprehensively in the areas related to information needs, services, emotional and social consequences and health problems and related issues (Annexure 1). Each item in this tool has a “Yes/No” response and a “Yes” indicates that the need is present and unmet; a response of “No” indicates either that the need is not present or that the need has been met. A three-phase process followed as proposed by the article on best practices for developing and validating scales for health, social and behavioural research [17].

### Phase 1- item development- cultural adaptation of the items ensuring content validity

As this tool was developed in the United Kingdom it was required to undergo cultural adaptation and modifications. The modified Delphi technique with an iterative process was carried out in two rounds to culturally adapt the tool [18]. A group of experts from the fields of Rheumatology and Rehabilitation, Neurology, General Medicine, Family Medicine, and Community Medicine was invited to review each item in the questionnaire and to indicate whether each item should be retained. If they decided to retain an item, then they assessed the cultural appropriateness of the words and examples using a 1–5 scale. An average score of 4 or above was taken as an agreement of the experts on the cultural appropriateness of an item. If the average score of an item was less than 4, the modifications suggested by the experts were reviewed. The agreement of more than 50% of the expert panel to remove an item, was taken as the cut-off to remove the item. Additionally, they were requested to indicate any additional items they think were relevant. The communications were made via individually addressed electronic mail. The identity of individual panellists was not revealed to the others until the end of the process.

### Phase 2- scale development – translating and pretesting

Translation and Pretesting of the culturally adapted LUNS tool.

Considering the likely varying levels of educational backgrounds and the recovery of patients, we adapted the tool to be used as an interviewer-administered tool.

The forward-backward translation method was employed in the translation of the adapted LUNS monitoring tool. The questionnaire was translated in to Sinhala with an emphasis to ensure semantic equivalence, conceptual equivalence and normative equivalence by two independent translators, both with a high level of proficiency in English and Sinhala. Sinhala-translated versions were then reviewed by an independent expert and with the suggestions from the expert Sinhala translated the version, again back translated by two other bilingual translators, independently without referring to the original English version. The two English translated versions were then reviewed by an independent expert, proficient in both English and Sinhala languages. The translated versions of the tool were finalized with the comments from the independent expert.

Pretesting of the Sinhalese version of the LUNS monitoring tool was conducted among ten stroke survivors who had completed approximately six months of the post-stroke period at the medical clinic at the District General Hospital Matara of the Southern province of Sri Lanka.

### Phase 3- scale evaluation – testing validity, reliability and overall acceptability

A) Establishing factorial validity.
  As the factorial validity of the LUNS tool is not established in many different settings elsewhere, it was decided to carry out Exploratory Factor Analysis (EFA) using Principal Component Analysis (PCA) to explore the factor structure of the tool. This analysis helped in identifying tools that represent single domain or multiple domains as expected with the multidimensionality of the unmet need concept. A descriptive cross-sectional study was conducted in the Teaching Hospital- Karapitiya of the Southern Province, for the validation study. Since the Sinhalese version of LUNS tool only having 21 items, indicating that the required minimal sample size was 105 (21 × 5) for the exploratory factor analysis [19]. With an addition of 15% to account for non-response, the required sample size was estimated at 120. A descriptive cross-sectional study was conducted in the Teaching Hospital- Karapitiya of the Southern Province, for the validation study. The required number of 120 study units was allocated to be recruited equally from the clinic held on each day of the week and eligible study units were recruited consecutively. The study population consisted of an adult population with a diagnosis of stroke and had completed six months from the time of the acute stroke incident. Stroke survivors who are resident of the Western province, and who cannot communicate due to ill health or who cannot communicate in Sinhala were excluded from the study. The study sample was described using socio-demographic characteristics such as numbers and percentages.
  Before EFA, factorability of the data was assessed using Bartlett’s test of sphericity. The Kaiser-Meyer-Olkin test was performed to measure the adequacy of the sample size to proceed into a factor analysis. Factor structure and factor loadings after varimax rotation were assessed. The items were observed for their factor coefficients and more than 0.4 were considered as well loaded. The items which could be grouped were identified by the factor coefficients of each item. In conjunction with the above criteria, the factors that lead to a meaningful interpretation and theoretical sense were ultimately selected.
B) Testing convergent and discriminant validity.
  Convergent and discriminant validity of the Sinhalese version of the LUNS monitoring tool was done by comparing it with the results of the Barthel Index (BI) and General Health Questionnaire 12 (GHQ-12). Calculating correlation coefficient by using quantitative results of the BI and GHQ-12 against the number of unmet needs estimated by using the LUNS tool [20].
  BI is a commonly used validated tool to assess activities of daily living of patients with physical impairments [21]. This tool is validated throughout the world for different settings and different languages. These studies reported high Cronbach alpha values for internal consistency was above 0.85 and inter-class correlation for interrater reliability was above 0.903 for the Barthel Index [22–25]. The original tool was developed for a total score ranging from 0 to 100 for 10 items, but new scoring system was introduced in 1988, where scores range from 0 to 20 [22]. BI with a modified version of scores, validated in Sri Lanka [26]. GHQ-12 is a globally recognized validated tool to assess psychological distress [27]. It is a 12-item questionnaire that can be self- administered within a very short period. Many studies had confirmed its reliability, external validity and factor structures in different settings with different languages too [28–32]. This tool was validated in Sri Lanka [33].
  It was expected that a high number of unmet needs was interrelated to a high GHQ 12 score and high number of unmet needs was interrelated to a low Barthel Index value. Based on this estimate the required number of subjects for a power of 80% and a two tail α of 0.05 was 85. With an addition of 10% to account for non-response the required sample size was estimated at 94. Since the assessment of EFA and discriminant validity, were conducted simultaneously, the final sample size was taken as 120. Scoring of the Barthel Index and GHQ 12 study instruments was carried out according to the instructions provided to assess the convergent and discriminant validity [20].
C) Testing reliability.
  The overall reliability index was assessed using Cronbach’s alpha coefficient of the Sinhalese version of the LUNS monitoring tool on the data of the validation study [16]. Internal consistency estimates of a magnitude of 0.70 or greater were considered satisfactory [17].
D) Appraising the acceptability of the Sinhalese version of the LUNS monitoring tool.
  This was measured by estimating the response rate for each item of the Sinhalese version of the LUNS monitoring tool in the validation study and the time duration taken to complete the tool.

## Results

### Phase 1- scale development- cultural adaptation of the items ensuring content validity

The results of the first round of iteration showed that more than 50% of the Delphi participants were indicating the removal of item numbers 2, 11, 13, 19 and 22. The mean scores for the appropriateness of the words and examples used for other items ranged from 2.8 to 5.0. Items 03, 06, 07, 08, and 20 had a mean score of less than 4.0 indicating that the words or examples should be modified to make them more culturally appropriate (Table 1). Experts have indicated that item number 6 is required to be modified as two separate items, while items 3, 7, 8 and 20 were suggested to be modified.

**Table 1:** Modifications to items of the LUNS tool according to the suggestions by the expert panel

Item number	Original item	Modified item
3	I regularly get pain and nothing seems to ease it	I regularly get pain and need further medications for relief
6	I need additional aids (e.g. kitchen equipment) or adaptations (e.g. stair lift, grab rails) inside the home	I need additional special aids to household activities
		I need some changes in side my home according to my needs
7	I need adaptations outside the home (e.g. ramp, rail) but they haven’t been ordered yet or I’ve been waiting too long	I need adaptations outside the home
8	I need some help / advice about getting back to driving and / or getting a blue badge	I need some help in getting back to driving
20	I often feel quite low, angry or worried and would like to find out what help is available	I need help in control my anger

More than half of the experts suggested including unmet needs related to sleep problems, hearing issues and vision problems also to the tool. A Modified tool was used during the second iteration and the expert panel to re-rate the cultural appropriateness of the words used in the modified items on a scale of 1–5. The scores for the second round were summarized and it was shown that the mean score of all the items was above 4.0 indicating that experts accepted this version as a culturally appropriate version of the 21 item LUNS monitoring tool to be used in the Sri Lankan setting to assess unmet needs of the strike survivors in the post-stroke period.

### Phase 2- scale development – translating, pretesting and establishing factorial validity

A) Pretesting of the culturally adapted LUNS tool.
  Based on the responses provided changes made to the instructions for data collectors and end users confirmed the questions and answers were meaningful.
B) Establishing factorial validity.
  The socio-demographic characteristics of the descriptive study conducted for establishing factorial validity are shown in Table 2. A total of 119 stroke survivors were recruited for the validation study. None were cognitively impaired. The mean age of the population was 66.93 years (SD ± 12.3 years) and 54.6% (*n* = 65) of the study units were employed at the time of the stroke.

**Table 2:** Distribution of the validation study sample by socio-demographic characteristics

Socio-demographic characteristics	***N***=119	
	n	%
Age categories (in completed years)		
0-20	1	0.8
21-40s	2	1.7
41-60	28	23.5
61-80	74	62.2
Above 80	14	11.8
Sex		
Male	73	61.3
Female	46	38.7
Highest level of Education achieved		
Never gone to school	33	27.8
Grade 1-5	35	29.5
Grade 6-11	21	17.6
Passed GCE O/L	6	5.0
Passed GCE A/L	3	2.5
Graduate/ Diploma Holder	2	1.7
Post-Graduate	1	0.8
Other	18	15.1
Type of stroke		
Hemorrhagic	19	15.9
Ischemic stroke	100	84.1

The presence of unmet needs among the stroke survivors who participated in the validation study is describes in the Table 3. The need for information related to stroke, diet and social benefits was very high among the study population. Approximately 1/3 of the study population still requires needs related to activities of daily living. The need for information related to the continuing the sexual activities was only reported by 3.4% (*n* = 4) and it’s the lowest unmet need declared.

**Table 3:** Distribution of unmet needs among the study population (*N* = 119)

Question	Presence of unmet need	
	n	%
1- I need more information about my stroke (e.g. what is a stroke, why it has happened to me and how to avoid having another one)	116	97.5
2- I need more information on my diet (e.g. alcohol, sugar, fat and salt intakes)	116	97.5
3- I need some help/ advice in choosing suitable transport	58	48.7
4- I need to know more information about social benefits available	103	88.0
5- I need further mediation to relive my pain	53	46.1
6- I need more help to control my bladder and bowel	10	8.5
7- I need help in getting better sleep	35	29.4
8- I need to help to have a better vision	31	26.1
9- I need help to hear better	7	6.2
10- I need help to stop me from falling	41	35.3
11- I need help in walking	37	32.2
12- I need additional aids to perform day to day activities (e.g. kitchen equipment) or adaptations (e.g. stair lift, grab rails) inside the home	36	30.8
13- I need more help with things like cutting my toenails, washing myself or dental care (including dentures)	25	27.7
14- I need help in maintaining and cleaning house, washing laundry, cooking, ironing at home.	36	30.3
15- I need adaptation/ modifications inside the house	44	37.0
16- I need adaptations outside the home (e.g. ramp, rail	44	37.0
17- I need more information on continuing sexual relationships	4	3.4
18- I need help in control my anger	44	37.3
19- I need some help / advice about getting back to driving a vehicle	7	5.9
20- I need more advice/Training on suitable employment	45	38.1
21- I need help to occupy my day better	77	65.3

The Kaiser-Meyer-Olkin test was performed, and it was 0.782 which is greater than the recommended minimum value for factor analysis. Bartlett Test Statistics was 1798.548; *p* < 0.001. Above data confirmed that the data set was acceptable for factor analysis.

The communalities of the items against the items of the Sinhalese version of the LUNS monitoring tool conducted and the question item number 3 and 4 dropped from the factor analysis due to communality values less than 0.5 level. We performed the factor analysis and varimax rotation in the principal component analysis revealed 6 factor structure. This was confirmed with oblique rotation method as well. However, we realised a single question loaded as a single factor (Item 19- driving). Therefore, we repeat analysis with the option of fix number of factors as 5, and it revealed question 19 loaded with questions 5 and question 7. Following consultation with an expert in the field, research group decided grouping above 3 items together is not appropriate and therefore question 19 also removed from the final analysis.

Table 4 shows the factor coefficients of individual items after Varimax rotation in the PCA procedure mapped with the domain structure of the LUNS tool after removing questions 3,4,19.

**Table 4:** Rotated component matrix using varimax rotation

Question No	Component				
	1	2	3	4	5
Question 1			0.962		
**Question 2**			**0.964**		
**Question 5**					**0.786**
**Question 6**		**0.656**			
**Question 7**					**0.768**
**Question 8**		**0.549**			
**Question 9**		**0.805**			
**Question 10**	**0.899**				
**Question 11**	**0.894**				
**Question 12**	**0.956**				
**Question 13**	**0.877**				
**Question 14**	**0.950**				
**Question 15**	**0.946**				
**Question 16**	**0.930**				
**Question 17**		**0.809**			
**Question 18**				**0.588**	
**Question 20**				**0.815**	
**Question 21**				**0.814**	

Extraction Method: Principal Component Analysis. Rotation Method: Varimax with Kaiser Normalization and rotation converged in 5 iterations

To confirm the factor structure, exploratory factor analysis was also repeated using the oblique rotation method and it also confirmed the same factor structure. Therefore, EFA was concluded with 3 questions (question no − 3, 4, 19) reduced and confirmed the construct validity of the Sinhalese version of LUNS monitoring tool.

We named component 1 (Questions 10,11,12,13,14,15,16) as needs related to activities of daily living, component 2 (questions 6, 8,9,17) as needs related to sensations, component 3 (questions 1,2) information needs, component 4 (questions 18,20,21) as needs related to productive life, component 5 (questions 5,7) as needs related to pain.

### Phase 3- scale evaluation – testing validity and reliability and acceptability

A) Construct validity- convergent and discriminant validity.
  The normality of the data was assessed using the Kolmogorov-Smirnov and Shapiro-Wilk test and it revealed data had a non-normal distribution. Hence, a non-parametric test, Spearmen r was applied to estimate the correlation between the number of unmet needs and scores for GHQ 12 and the Barthel Index [20]. Results of the Spearmen r against the GHQ_12 and Barthel Index are shows in Table 5.
  Test results revealed that both GHQ12 and Barthel Index significantly correlated as expected with number of unmet needs among stroke survivors.
B) Results of the reliability assessment.
  The internal consistency of the LUNS tool was assessed by calculating Cronbach’s alpha. The overall Cronbach’s Alpha value was 0.877.
C) Appraising the overall acceptability.
  The mean time taken to complete the Sinhalese version of the LUNS monitoring tool section of the questionnaire was assessed and showed that it was 17.52 min (SD = 2.72). The response rate for each item of the Sinhalese version of the LUNS monitoring tool was also estimated to measure the acceptability and the overall response to all the items was above 98.93%, confirming acceptability.

**Table 5:** Correlation between unmet needs with GHQ 12 and barthel index

		Total Unmet needs
GHQ_12	Correlation Coefficient	.403^a^
	Sig. (2-tailed) (*P*<0.05)	.000
	N	119
Total_BI	Correlation Coefficient	-.687^a^
	Sig. (2-tailed) (*P*<0.05)	.000
	N	119

^a^Correlation is significant at the 0.05 level (2-tailed)

## Discussion

The unavailability of a validated tool to measure the unmet needs of stroke survivors in Sri Lanka, led to the validation of the LUNS tool to measure unmet needs among stroke survivors at the end of 6 months post-stroke period. The use of the modified Delphi technique instead of face to face consultative meetings facilitated the independence of forming opinion and perspectives as it prevented the manipulation of opinion by influential individuals in culturally adapting the LUNS tool [18]. Two items on needs for transport and information on social benefits were removed since the majority of the stroke survivors were elders, and they were looked after by known caregivers, who take care of patients’ transport and social benefits in Sri Lanka. Therefore, we tested 21 items modified tool opposed to 22 items validated by the original study.

We modified the LUNS tool to be administered as an interviewer-administered tool compared to its original development in the UK and subsequent adaptation in the Netherlands [14, 16]. We adopted this approach considering the varying level of literacy, response rate and availability of its results in a short time enable the healthcare team to adjust treatment and rehabilitation as per individual stroke survivor needs. Concerning the percentage of unmet needs reported in the validation studies, in the Netherlands only 46.2% (*n* = 36), declared information on stroke, whereas in our study it’s 97.5% (*n* = 116), but this highest declared unmet needs among study participants [16]. This is probably due to the greater involvement of the social support worker schemes in the Netherlands compared to Sri Lanka or this could be the period between stroke incidence and study is much longer (5–8 years vs. 6 months). Additionally, medication requirements in the Netherlands study were reported as 16.7% (*n* = 13), compared to 46.1% in our study. These large numbers could also be attributed to our stroke survivors still in the early phase of the recovery but also could be due to lack of comprehensive care associated observed in Sri Lanka for stroke follow-up care. Like our study, the lowest reported unmet need among the Dutch population also the advice on a physical relationship (3.8%, = 3) could still be related to any stigma associated with declaring or in general study participants are elderly age group and lack of interest in it.

In our study, we identified possible 5 underlying factors through exploratory factor analysis. The items related to activities of daily living of component 1, and 2 items loaded into information needs of component 3 shows high internal consistency value within the component, displaying validity of the grouping. Four items were loaded into component 2, but we believe the need for information related to continuing sexual relationships should have been categorized with component 3. The items of need help in controlling anger ideally should have been loaded with component 2 as it is more related to sensory needs. Furthermore, 2 items of component 5 related to pain relief and sleep may have been loaded with the needs of component 1, as these could be the result of physical health issues. The development team for the LUNS tool in the UK conducted a confirmatory factor analysis on one dimension but failed. Their exploratory factor analysis revealed a possible 3 or 4 factor structure, but they have not described items under each component [34]. The study conducted in the Netherlands also did not report on factorial validity. Evaluation of concurrent validation with BI and GHQ-12 revealed that unmet needs had a moderate and significant correlation with both BI (*p* < 0.001) and GHQ-12 (*p* < 0.001) confirming its validity. GHQ − 12 was used in the UK to assess concurrent validity and similar to UK study, we identified that high unmet needs had significantly poorer health scores for GHQ-12 (*p* < 0.05) [14]. They also reported Frenchy Activities Index (FAI) significantly correlated with poor health status. As FAI was not validated in Sri Lanka, we used BI, which has 10 activities compared to 15 items in FAI. We believe BI is still good enough to assess the trend of correlation between unmet needs and BI scores. As expected, high unmet needs correlated with poor BI values as well. The Netherlands study also used FAI to assess the concurrent validity, and they found that out of 22, 15 items had an association with FAI [16]. Both the UK and Netherlands studies additionally used the Short Form − 12, Physical and Mental component tools, but we did not use SF-12 as we believed components assessed already covered by BI and GHQ-12 and SF-12 also not been validated in Sri Lanka yet. The major difference in the Dutch study is that it was conducted among stroke survivors of 5–8 years duration compared to Sri Lanka and UK studies where it was 6 months duration.

The overall Cronbach’s alpha value for 19 items of the LUNS tool in our study was revealed as 0.877 revealing good inter-rater reliability while the reliability assessment of the original LUNS has been reported as 0.815 [14]. However, in adapting this tool to the Netherlands Cronbach’s alpha values were not reported [16].

The high response rate (98.93%) with an average completion time of 17 min confirmed that the LUNS tool can be adopted in Sri Lankan outpatient department clinic set up in assessing the follow up. In comparison with an adaptation of this tool to the Dutch population, the study team performed this study on stroke survivors with 5–8 years duration [16]. They also followed the forward and backward translation together with revisions from experts. However, they have used the original 22 items probably due to cultural and socio-economic similarities between the Netherlands and the United Kingdom when compared to developing countries like Sri Lanka.

There were some limitations to the study. We adopted the modified Delphi technique in culturally adapting the LUNS tool to Sri Lanka with a multidisciplinary team. However, if we could have conducted a consensus meeting for this purpose might have developed better results in culturally adapting the tool. A potential source of selection bias was that the validation only included stroke survivors attending the hospital clinics and their unmet needs are likely to differ from the unmet needs of stroke survivors who do not attend follow up clinics. We could not conduct test-retest validation and it could have added additional evidence related to the validation of the tool. As there is no gold standard methodology to assess the unmet needs, we used BI and GHQ-12 to assess the concurrent validity as these were already validated in Sri Lanka. However, the use of FAI and SF-12 could elicit better results than the UK and Netherlands studies. Participants were recruited from one hospital in a province, which may affect the generalizability of the study findings. Some of the information related to unmet needs are considered culturally sensitive in nature and therefore utilizing the interviewer-administered questionnaire might lead to some underreporting. As far as possible the stroke survivor him/herself was used as the respondent since unmet needs are best known to self and the sensitive nature of the items being inquired into. Although we used a brief tool to assess the cognitive status of the study participants, there may have been some level of cognitive impairment undetected with the brief tool, which could have had impact on their responses to the interviewer-administered questionnaire.

## Conclusion

The evidence suggests this tool can be adapted to use in Sri Lanka to assess unmet needs among stroke survivors. However, we recommend further studies to assess its validity and reliability among a wider group of stroke survivors.

## Data Availability

The datasets used and/or analysed during the current study available from the corresponding author on reasonable request.

## Abbreviations

EFA: Exploratory Factor Analysis
GHQ-12: General Health Questionnaire
LUNS: Longer-term Unmet Needs after Stroke
PCA: Principal Component Analysis
